# Hospital Facebook Reviews Analysis Using a Machine Learning Sentiment Analyzer and Quality Classifier

**DOI:** 10.3390/healthcare9121679

**Published:** 2021-12-03

**Authors:** Afiq Izzudin A. Rahim, Mohd Ismail Ibrahim, Sook-Ling Chua, Kamarul Imran Musa

**Affiliations:** 1 Department of Community Medicine, School of Medical Science, Universiti Sains Malaysia, Kubang Kerian, Kota Bharu 16150, Kelantan, Malaysia; drafiqrahim@student.usm.my (A.I.A.R.); drkamarul@usm.my (K.I.M.); 2 Faculty of Computing and Informatics, Multimedia University, Persiaran Multimedia, Cyberjaya 63100, Selangor, Malaysia

**Keywords:** health informatics, machine learning, topic classification, sentiment analysis, Facebook, SERVQUAL, Malaysia

## Abstract

While experts have recognised the significance and necessity of social media integration in healthcare, no systematic method has been devised in Malaysia or Southeast Asia to include social media input into the hospital quality improvement process. The goal of this work is to explain how to develop a machine learning system for classifying Facebook reviews of public hospitals in Malaysia by using service quality (SERVQUAL) dimensions and sentiment analysis. We developed a Machine Learning Quality Classifier (MLQC) based on the SERVQUAL model and a Machine Learning Sentiment Analyzer (MLSA) by manually annotated multiple batches of randomly chosen reviews. Logistic regression (LR), naive Bayes (NB), support vector machine (SVM), and other methods were used to train the classifiers. The performance of each classifier was tested using 5-fold cross validation. For topic classification, the average F1-score was between 0.687 and 0.757 for all models. In a 5-fold cross validation of each SERVQUAL dimension and in sentiment analysis, SVM consistently outperformed other methods. The study demonstrates how to use supervised learning to automatically identify SERVQUAL domains and sentiments from patient experiences on a hospital’s Facebook page. Malaysian healthcare providers can gather and assess data on patient care via the use of these content analysis technology to improve hospital quality of care.

## 1. Introduction

Public health professionals need accurate and up-to-date data from a range of sociodemographic categories to develop effective quality management systems for healthcare services and policy activities. Patient satisfaction is a critical indicator of the quality of care provided in a hospital environment [1,2,3]. By recognising and comprehending the elements that influence patient perceptions, healthcare practitioners may more effectively meet or surpass patient demand for high-quality treatment [4].

To assess patient satisfaction with various aspects of service quality, patient satisfaction surveys such as the Hospital Consumer Assessment of Healthcare Providers and Systems (HCAHPS) and service quality (SERVQUAL) questionnaires are frequently used [5,6,7,8]. These surveys are the product of years of assessment, are methodical in their administration and review, and may gather many patients’ replies per institution [9,10,11]. Nonetheless, they are expensive to administer, time-consuming, have low response rates, require considerable time between hospitalisation and public publication of results, frequently fail to identify the underlying cause of reported problems, and are subject to selection and response bias [5,6,12,13]. The discrepancy between the traditional patient survey and other data sources highlighted the need to use other data sources to assess public opinion on healthcare services [14]. As a result, the internet and social media have been recommended as potential substitutes for assessing patient satisfaction and evaluating the quality of healthcare services [15,16].

There is increasing recognition that user-generated information available via social media platforms such as Facebook, Twitter, and Yelp may be a significant source of data for patient experience and quality-of-care measures [17,18]. This data may be used to enhance and broaden the breadth of patient experience and health quality services by strengthening their early warning monitoring capabilities for healthcare quality management [19,20]. However, social media presents a slew of problems for data collection and analysis in online settings relevant to healthcare research. To begin, conversations on social media platforms may devolve into a range of subjects, not all of which are necessarily linked to healthcare [5]. Second, in contrast to the structured data contained in electronic medical records or clinical notes written by healthcare providers, patient feedback on social media is frequently expressed in unstructured text, necessitating the detection and extraction of interpretable factors for improved comprehension [21]. Third, it is often necessary to infer the quality of the users’ therapy or clinical results from their evaluations [13].

While this may be achieved manually through human input, such processes are often inefficient and time-consuming [22,23]. Another option is to use crowdsourcing to expedite the process, but this can be quite costly (domain experts, for example, are expensive in terms of expertise and time, and the cost typically varies according to the number of tasks assigned), and in some cases, privacy concerns require sharing such data with contractors and consultants. Automated approaches, often based on machine learning (ML), are being progressively used to overcome these barriers.

In Malaysia, an annual patient satisfaction survey is conducted using the SERVQUAL method in public clinics and hospitals [24,25]. However, as previously said, it has several disadvantages. To continually enhance the quality of service and patient satisfaction, machine learning algorithms must be developed to augment traditional outcomes and support healthcare stakeholders in making timely choices. The purpose of this study is to design and assess the performance of machine learning quality classifiers (MLQC) and machine learning sentiment analyzers (MLSA) in automatically identifying SERVQUAL dimensions and sentiments in Facebook reviews of Malaysian public hospitals.

## 2. Related Works

### 2.1. SERVQUAL and Social Media

The SERVQUAL model is a widely used approach for evaluating the quality of service in a variety of service contexts, sectors, and nations [26]. The technique makes it simple to assess both customer service requirements and customer service perceptions [27,28]. The creation of the SERVQUAL scale revealed five dimensions of perceived quality: tangibles, reliability, responsiveness, assurance, and empathy. The “tangibles” dimension encompasses all physical aspects of the service quality experience (e.g., equipment, facilities, personnel). The terms “reliability” and “assurance” refer to consumers’ perceptions of a service provider’s ability to provide the service. The former involves assessing the service provider’s reliability and accuracy, while the latter involves assessing the service provider’s attributes such as knowledge and courtesy, which may inspire trust and confidence in the provider. The “responsiveness” component is concerned with the perceived helpfulness and promptness of the service provider. Finally, the component referred to as “empathy” pertains to how individuals perceive personalised, caring service [28].

The SERVQUAL model has been used to evaluate service quality in hospitals and healthcare settings, mostly using survey-based methods. Numerous studies conducted in Malaysia have established and validated the SERVQUAL model for measuring the quality of healthcare services [24,25,29,30]. SERQUAL and other quality measures are the product of years of assessment, are conducted and analysed in a systematic manner, and have the potential to gather many patient answers per institution [9,31]. Nonetheless, the surveys have several drawbacks, including being costly to administer, time-consuming, requiring significant time between hospitalisation and public publication of results, frequently failing to identify the underlying cause of reported problems, and being subject to selection and response bias [5,6,13]. The contrast between typical patient surveys and real-time public opinion about healthcare services highlights the need for additional data sources for analysing real-time public opinion about healthcare services [14]. Therefore, the internet and social media platforms have been proposed as a new method of reviewing and monitoring the quality of healthcare services [12,15,16,32].

However, social media data is often massive and presents a range of challenges, including data cleaning, data processing, and developing a theoretical model of social media content quality. While this may be accomplished manually by human input, the process is lengthy, and the method’s validity and reliability are often questioned. A systematic review of patient online reviews established and recommended the use of advanced analytical methods such as machine learning to accelerate the processing of vast amounts of online review data [13]. Monitoring service quality using hospital social media platforms may assist all stakeholders in recognising quality issues and minimising the need for expensive and time-consuming surveys. Despite their uncommon, research on Facebook content analysis demonstrates a link between social media quality categories and traditional quality assessments [33,34,35,36].

### 2.2. Machine Learning, Sentiment Analysis, and Topic Classification

Apart from finance and marketing, machine learning has been used in clinical medicine and healthcare improvement on a regular basis. Machine learning has been used in patient care [37], stroke prediction [38], cardiology [39], and personal health investigations [40]. Additionally, machine learning is used to quantify patient experience input, which is often achieved by sentiment analysis and text classification [22,41]. Social media sentiment analysis is advantageous for assessing how people feel about goods, events, people, and services. It employs word patterns to determine if a statement in patient feedback is a complaint or a compliment. This automated process helps healthcare organisations by delivering findings faster than a human strategy would [42]. Meanwhile, topic or text analysis is a technique for analysing vast amounts of unstructured data in order to elucidate the text’s primary subjects [43]. Social media data had the same enormous potential for researching health quality issues or themes as a validated and established traditional survey [33,44].

The two most commonly used approaches for text and sentiment analysis were supervised and unsupervised learning [22]. The approach that was most often employed was supervised learning, which involves manually categorising a subset of data according to themes and sentiment [45]. Comprehensive reading of all comments included inside the dataset continues to be the “gold standard” approach for free text comment analysis, since it is the only way to assure that all relevant comments are coded and analysed [22]. In supervised learning, the most often used classifiers are SVM and NB, both of which consistently exhibit high classification performance. In structured patient surveys, a supervised approach is often used to analyse online reviews [5,46,47]. On the other hand, topic modelling is an unsupervised machine learning technique that makes use of Latent Dirichlet Allocation (LDA) to automatically identify topics within a given remark [48]. LDA is a text generation model based on the premise that the words in a document represent a collection of latent themes (each word relates to a specific subject). Apart from identifying the most discussed themes in individual comments, a topic model may be utilised to find fresh insights within the free text. Consequently, this technique is often used to analyse unstructured social media comments [49,50,51].

Metrics like accuracy, sensitivity, recall, specificity, precision, hamming loss, and the F-measure may be used to assess machine learning performance. The model’s F1 score indicates its quality [52]. In a machine learning performance evaluation of cancer treatment experience, the SVM algorithm had the highest overall sensitivity (78%), accuracy (83.5%), and overall f-score of 80% in sentiment analysis [53]. As shown in the RateMD website research, sentiment analysis using the NB classifier has a positive score of 0.94 and a negative score of 0.68, with an average score of 0.825 for text classification [46]. Meanwhile, a study of patient satisfaction at the Utah Health Care System discovered a sentiment score of 0.84 and a text score of 0.74 when the NB classifier was used [43]. Another research indicated that using the NB algorithm, patient tweets from the English National Health Service (NHS) had a sentiment score of 0.89, a theme score of 0.85 for dignity and respect, and a text classification score of 0.84 for cleanliness [47]. However, a machine learning sentiment analysis of Facebook comments using the SVM approach obtained an F1 score of 0.87 [54], equal to an average of 0.89 and 0.84 in topic classification studies of NHS tweets [5,55]. The findings indicate that SVM and NB may be used interchangeably as preferable classifiers in a supervised setting since they outperformed other classifiers in sentiment analysis and text classification.

## 3. Materials and Methods

### 3.1. Facebook Data Collection

This research analysed data collected from Facebook reviews that were publicly accessible on official hospital Facebook pages between January 2017 and March 2018. We collected all 1793 Facebook reviews from 48 official Facebook pages of public hospitals in Malaysia. WebHarvy software (SysNucleus, Kochi, India) was used to extract the data. All collected reviews were manually checked and any irrelevant reviews, such as business promotion or marketing, or reviews from hospital departments’ Facebook pages or from the pages of health institutions or agencies such as the Ministry of Health (MOH), the Institute of Medical Research (IMR), non-governmental organisations (NGOs), and long-term care facilities were excluded. Malaysia is a multiracial nation with a diverse range of languages and dialects. Our national language is Malay, while English is our second language. As a result, we collected reviews exclusively in those languages. After harmonising the dual-language Facebook data into a standard language, the Malay language data was translated into English manually by local junior doctors to ensure appropriate translation.

### 3.2. Development of Machine Learning Quality Classifier (MLQC)

Manual coding was employed to create a labelled data set that would serve as a “gold standard” for machine learning quality classifiers (MLQC). The term “classifier” refers to the class labels applied during the manual annotation phase that the machine classification models attempt to accurately label [33]. To begin, two hospital quality managers or SERVQUAL model specialists were hired to perform a preliminary “open” coding on multiple batches of 100–300 Facebook reviews based on the MOH SERVQUAL patient satisfaction survey to establish the source coding guidelines (Appendix A Table A1). We also used the survey items of other SEVRQUAL studies to enhance the descriptions in the corresponding dimensions. Then, a random subsample of 300 Facebook reviews was chosen to test intercoder reliability. The raters separately coded the reliability subsample using Microsoft Excel. For each SERVQUAL dimension, Cohen’s Kappa values were utilised to determine in-ter-rater agreement. Coding of Tangible (Cohen’s = 0.885, *p* < 0.001), Empathy (Cohen’s = 0.875, *p* < 0.001), Reliability (Cohen’s = 0.736, *p* < 0.001), and Responsiveness (Cohen’s = 0.72, *p* < 0.001) characteristics from Facebook evaluations exhibited high agreement, but agreement for Assurance (Cohen’s = 0.626, *p* < 0.001) was modest. Cohen’s coefficient was 0.769 on average for all dimensions. The sample of 900 manually labelled Facebook reviews were used to train our MLQC tool.

The machine learning technique examines the characteristics of the individual phrases used in the Facebook reviews and uses this data to build a quality domain classifier. Firstly, the labelled dataset was preprocessed by eliminating URLs, numerals, punctuation marks, and stop words, as well as by reducing words to their base forms using a lemmatization technique (e.g., treating as treat). Following that, we utilised the term frequency-inverse document frequency (TF-IDF) technique to determine the weight of terms, which indicates their significance to the documents and corpus. For each term t(i) in a Facebook review j, the TF-IDF score was computed as w(i, j) = tf(i, j) × idf(i). The term frequency tf(i, j) refers to the number of times a term t(i) appears in a Facebook review j. The idf(i) is the inverse document frequency, which equal to log(N/df(i)) where N denotes the total number of Facebook reviews in the dataset and df(i) is the number of Facebook reviews that include term t(i). Each Facebook review is expressed as a feature vector, with each item representing the feature’s TF-IDF score.

**Figure 1:**
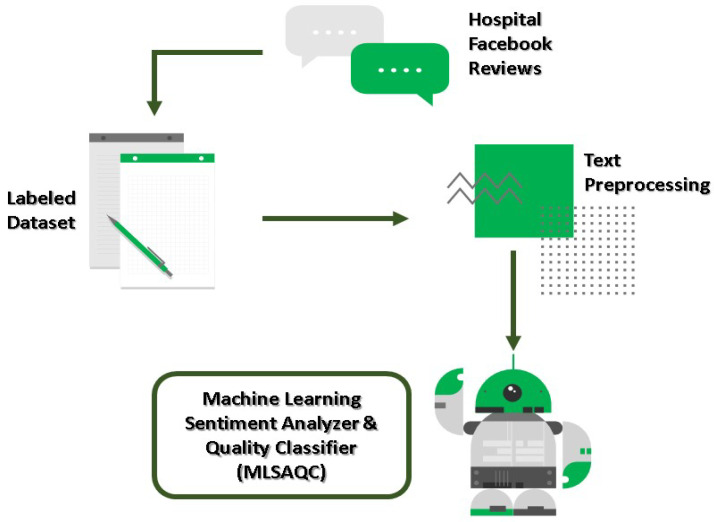
Machine Learning Development Process.

Different multi-label techniques were trained for topic classification, including Binary Relevance, Label Powerset, Classifier chain, RAkEL: RAndom k-labELsets, ML-KNN: Multi-label k-Nearest Neighbor, and BRkNN: Binary Relevance k-NN. These multi-label techniques are applied to transform multi-label problems into one or more single-label problems. With such a transformation, it allows us to apply single-label classifiers. For each technique, we trained three base classifiers: Naive Bayes (NB), Support Vector Machine (SVM), and Logistic Regression [1]. NB, SVM, and LR are all widely used classification methods that have been demonstrated to perform well on text classification tasks [42,52]. To ensure that all the quality labels are included in the training and test sets, we have applied iterative stratification sampling. The multi-label classifiers were evaluated using the Python software via the scikit-multilearn library [56]. There were studies that applied a similar approach to topic classification models [5,12,43,46,53]. The process of topic classification is summarised in Figure 1.

### 3.3. Development of Machine Learning Sentiment Analyzer (MLSA)

As with topic classification, we created a labelled data set for our machine learning sentiment analyzer (MLSA) using a manual coding approach. Again, our hospital quality managers, who are well-versed in-patient satisfaction surveys, were appointed to do open coding on 100–300 randomly selected Facebook reviews to generate a coding guideline (Table A2). After that, a randomly selected subsample of 300 Facebook reviews was used to assess intercoder reliability. The agreement between the coding of positive (Co-hen’s = 0.721, *p* < 0.001) and negative sentiment (Cohen’s = 0.686, *p* < 0.001) was satisfactory. However, the neutral or unidentified category of review had a lower degree of agreement (Cohen’s = 0.43, *p* = 0.027), which might be explained by the more amorphous and heterogeneous nature of this category. Thus, both quality managers will discuss and re-evaluate the neutral or unidentifiable group of sentiments. If the review stays neutral or unidentified, it will be eliminated, as we prefer to classify reviews using binary sentiment. In an earlier study, the binary technique has been verified and demonstrated to have superior accuracy, recall, and F-score performance when compared to multiclass sentiment classification (positive, negative, neutral) [57]. Following that, 1393 randomly selected data instances were tagged and preprocessed in preparation for machine learning training. For sentiment analysis, the training data is selected using a stratified sampling technique whereby 80% of reviews in each class are selected for training. Our machine learning model was trained using the Python software packages nltk, spacy, and scikit-learn based on three base classifiers: NB, SVM, and LR. A few techniques from previous studies were applied for sentiment analyzer development in this study [12,46,53,55]. Figure 1 illustrates the process of sentiment classification.

### 3.4. Machine Learning Performance Evaluation

A frequently used approach for the evaluation of classification algorithms is 5-fold cross validation, which minimises the bias in estimation of classifier performance [22,52]. This technique uses the labelled dataset for training and testing. Cross-validation applies to equal-sized selections of the manually labelled data set. The cross-validation procedure is rerun five times (the folds). Test data is always kept as a single subset, while the other four subsamples are utilised as training data. Once the results of 5 different folds are obtained, an average is computed for accuracy, precision, recall, and F-score. Precision is expressed as the ratio of accurately classified positive instances divided by the number of examples the model classifies as positive. Recall, often referred to as sensitivity, is the number of identified positive examples divided by the number of true positive examples in the manually coded data. The harmonic mean of precision and recall scores is an F-score. The higher the F1 score, the superior, with zero representing the worst conceivable result and one representing the finest possible result [22].

## 4. Results

### 4.1. Performance of Machine Learning Quality Classifier (MLQC)

**Figure 2:**
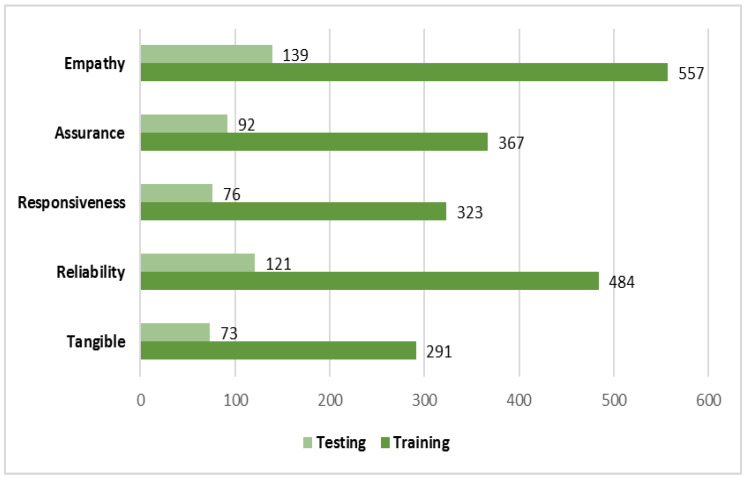
The number of records in training and test datasets for each SERQUAL domain.

The number of SERVQUAL domains in our training and testing sets is shown in Figure 2. Empathy has the most records, whereas tangible has the fewest. Table 1 summarises the prediction performance from the supervised machine learning, including the accuracy ratings for the highest performing classification model and multi-label classifier. Predictive performance ratings for classification models ranged between 0.13 and 0.25, indicating that the models correctly classified the reviews with an F1 value of 0.687 to 0.757. In comparison to other models and classifiers, overall, the SVM model with the classifier chain method has the highest accuracy (0.215) and F1-score (0.757). However, more importantly for the topic classification model is the hamming loss, which measures the fraction of class labels that are incorrectly predicted. The SVM model with a classifier chain has the lowest hamming loss (0.273) compared to other models. Meanwhile, SVM with the binary relevance method was the second best, after SVM with the classifier chain. All models were evaluated by 5-fold cross validation.

While our overall average accuracy was lower than that of prior supervised machine learning studies, the performance metrics for each SERVQUAL dimension demonstrated high predictive accuracy and an F1-score. The accuracy range for the tangible dimension was 0.635–0.740, the reliability dimension was 0.657–0.718, responsiveness was 0.536–0.718, assurance was 0.574–0.691, and empathy was 0.718–0.785. The F1-scores for tangible dimensions ranged from 0.388 to 0.624, dependability dimensions from 0.766 to 0.810, responsiveness from 0.404 to 0.655, assurance from 0.643 to 0.701, and empathy from 0.821 to 0.877.

Further examination of the Tangible dimension revealed that both the SVM model for binary relevance and the classifier chain had the highest F1-score (0.587). LR with binary relevance has the highest F1 score for the dimensions of reliability (0.823) and assurance (0.7232), while NB with label powerset has the highest score for responsiveness (0.633) and LR with label powerset has the highest score for empathy (0.886). However, only SVM with a classifier chain has a consistent high performance of an F1 score in all SERVQUAL dimensions. Therefore, the SVM model was used to train the machine learning quality topic classifier (MLQC) using the classifier chain technique. Table 2 summarises the performance metrics for each SERVQUAL dimension following 5-fold cross validation.

### 4.2. Performance of Machine Learning Sentiment Analyzer (MLSA)

**Figure 3:**
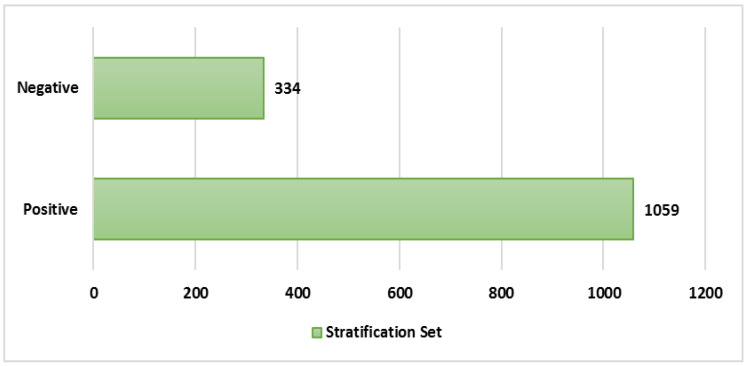
Number of Records used in Sentiment Analysis (n = 1393).

Figure 3 shows number of records split into positive and negative sentiment in this study. Overall, our binary sentiment classification revealed that SVM results outperform other machine learning techniques in terms of accuracy (0.874), precision (0.903), and F1-score (0.919) although NB has a higher recall (0.999). Meanwhile using hold out method, the SVM model still has the highest accuracy (90%) and F1 score of positive (93%) and negative (77%) sentiment compared to other ML models. Therefore, due to the high predictive accuracy and F1 score of the SVM model, we chose it for our machine learning sentiment analyzer (MLSA). Table 3 summarises the model evaluation following 5-fold cross validation and Table 4 describes results from hold out method.

## 5. Discussion

This is the first research to date in Malaysia to build a machine learning model for hospital quality of care monitoring. The results of this research show how supervised machine learning algorithms may be utilised to correctly classify SERVQUAL quality domain and sentiment-related content in Malaysian Facebook reviews. In this research, we demonstrate that SVM models with classifier chains outperform other models. Our findings almost replicated the performance of SVMs in classifying themes in a variety of experiments that used supervised machine learning and human classification. According to the RateMD research, SVM performance for staff-related topics was 0.85, whereas our score for empathy (like staff-related topics) was 0.88 using the same model [46]. According to an NHS Choice study, the subject of dignity and respect received an average score of 0.8, whereas cleanliness received an average score of 0.84 [47]. By comparison, the assurance dimension was 0.73 and the tangible dimension was 0.59 in our study. Nonetheless, their findings were validated just once or twice, as opposed to our 5-fold cross validation. Meanwhile, the overall performance of SVM-based topic classification in NHS Twitter research after 10-fold validation was 0.89, whereas our overall SVM model performed at 0.76 [5]. The benefits of having a large amount of data for analysis and a limited number of subjects for categorization are critical in determining the success of machine learning models. In comparison to the NB and LR models, our MLSA employing SVM model has a 0.92 accuracy after 5-fold validation, as well as the highest accuracy and F1 score in the holdout method, with 93% of positive and 77% of negative sentiment. In contrast to the RateMD research, they obtained 89% of positive and 64% of negative sentiments using SVM, whereas 94% of positive and 68% of negative sentiments were obtained using the NB model [46]. Additionally, the F1 score of sentiment analysis using the SVM model was between 0.80–0.87 in earlier research [43,47,53,54,55,58], indicating a higher F1-score in our work.

Combining two aspects of content analysis tasks, such as topic classification and sentiment analysis, is a new technique, especially in emerging countries with an expanding healthcare market and services. These findings suggest a mechanism for utilising the massive amounts of text on social media, and that further exploration of the information contained in free-text comments may be critical for understanding patient experience, supplementing traditional survey methods, and improving hospital quality management [13,52]. Another critical issue is that manual classification techniques will continue to be the de facto standard method for supervised machine learning analysis of patient online reviews [22]. Health is a complicated topic with a plethora of medical jargon, and each medical word has a distinct meaning. Health literacy and the presence of numerous languages complicate language analysis. As a result, thorough scanning of all comments is the only method to guarantee that all relevant opinions are coded and analysed correctly. This shows that machine learning-based language analysis is only as good as the training set used to guide it [12]. As a result, the experience and knowledge of coders or independent reviewers are critical for ensuring excellent machine learning performance using supervised learning [45,57,59]. Also, our research confirmed results from earlier comparable studies that SVM was the most commonly used classifier in supervised learning, followed by NB. SVM and NB have been extensively used for text and sentiment classification because they continuously perform well [22,42].

The study’s methodology allows policymakers to use social media sentiment about health care services as a substitute for conducting and scheduling more costly national questionnaire surveys. Also, because SERVQUAL serves as the foundation for public hospital patient satisfaction surveys in Malaysia, the conceptualization used in this study may serve as a supplement to the Ministry of Health’s hospital patient satisfaction survey and as a valuable early warning system for hospital quality management. Thus, via systematic monitoring of internet comments, we may discover societal views and integrate them into the design of high-quality healthcare services [19,20]. Furthermore, a systematic and effective strategy is needed to enhance the quality of the healthcare system. The proposal incorporates systematic, thorough monitoring and reporting of quality improvement initiatives, as well as a priority for responding to and learning from quality-of-care incidents [60]. To improve healthcare outcomes in Malaysia, it is necessary to collect data on patient online evaluations and to use systematic methods for evaluating patient feedback. However, they take a significant amount of time between hospital admission and report disclosure, often fail to identify the underlying causes of issues, and may introduce response and selection bias [5,13,47]. The difference between the traditional patient survey and other data sources underscored the significance of using alternative data sources to evaluate patient perceptions and views about healthcare services and to understand real-time patient management. Therefore, social media platforms are a good alternative for assessing patient satisfaction and evaluating the quality of healthcare services [16,32].

### Future Works and Limitations

Future studies should concentrate on improving sentiment analysis and topic classifier performance, as well as on collecting a bigger dataset of patient online reviews, including those from the private sector. Likewise, additional study is needed to expand the method’s application to other kinds of free-text content on social media. For example, various methods may be included to bolster the process, such as the assessment of unigrams, bigrams, or high n-grams, as well as the refining of contextual polarity [22]. Additionally, in future studies, neural network classifiers, deep learning algorithms, and Bert-based models will be explored and compared [23,38,52]. For example, a Deep Learning model built on Bidirectional Long-Short-Term Memory (LSTM) layers may be used to utilise cutting-edge vector representations of data, such as Word Embeddings [61]. Then, we can compare the outcomes of classical machine learning and deep learning approaches as performed in the previous study [62]. Also, it would be useful to compare the labelled dataset in this research to other dictionaries or tools used in previous studies to improve sentiment and text classification [41,63]. We are also interested in exploring other sampling methods to address the imbalanced data between the positive and negative reviews [64].

Numerous limitations apply to our research. Although supervised learning is time-consuming due to the human coding needed, it is useful for analysing patient online reviews that are often seen in structured surveys such as SERVQUAL and HCAHSP [54,57,63]. Owing to the increasing number of comments on social media, manual coding for supervised learning may become impractical due to time limitations. To address it, a topic modelling method based on latent Dirichlet allocation (LDA) may be beneficial in determining how closely the findings match what people with domain expertise have decided the subjects to be, as well as identifying new topics not previously recognised by humans [48]. Additionally, sentiment analysis and topic classification methods based on machine learning are only as successful as the training set used to guide them. However, our dataset is considered limited in contrast to other machine learning studies, because the use of social media reviews in the healthcare sector in Malaysia is still relatively new and Malaysia has a small population compared to the population studied in other similar research. Nonetheless, social media use in Malaysia continues to grow every year across all sociodemographic categories [65]. Thus, as is the situation in developed countries, we may expect an avalanche of social media user reviews of healthcare services. While our machine learning classifiers performed well, our study’s manual coding method presented the potential for selection bias. To reduce bias, we enlisted the assistance of two hospital quality managers who are acquainted with SERVQUAL domains and patient satisfaction surveys. Moreover, additional bias may exist since social media evaluations are usually produced by younger, wealthier people who reside in urban regions, although this prejudice was mitigated by including reviews from rural public hospitals.

## 6. Conclusions

By incorporating a manual coding approach into our supervised machine learning framework (MLSAQC), we proposed a strategy for auto-classification of SERVQUAL domains and sentiments on public hospital Facebook pages in Malaysia. The MLSAQC application will help healthcare providers by doing high-quality research, monitoring, and alerting them in real time to supplement other standard patient quality of care measurements in Malaysia.

## Figures and Tables

**Table 1:** Performance of ML models based on 5-fold cross validation.

Multilabel Classifier	Model	Accuracy	Recall	Precision	F1-Score	Hamming Loss
Binary	NB	0.147	0.761	0.701	0.730	0.315
Relevance	SVM	0.211	0.763	0.745	0.754	0.278
	LR	0.193	0.775	0.732	0.753	0.285
Label Powerset	NB	0.130	0.896	0.633	0.741	0.349
	SVM	0.166	0.799	0.679	0.734	0.323
	LR	0.158	0.825	0.669	0.739	0.326
Classifier chain	NB	0.149	0.756	0.705	0.730	0.313
	SVM	0.215	0.761	0.753	0.757	0.273
	LR	0.191	0.770	0.727	0.748	0.290
RakEL	NB	0.157	0.749	0.699	0.722	0.322
	SVM	0.186	0.764	0.724	0.743	0.295
	LR	0.180	0.765	0.726	0.745	0.293
MLkNN	N/A	0.140	0.737	0.697	0.715	0.327
BRkNN	N/A	0.157	0.648	0.732	0.687	0.330

**Table 2:** Performance metrics for each SERVQUAL dimension of MLQC following 5-fold cross validation.

Multi-Label	Base Classifier	Metrics	Tangible	Reliability	Responsive	Assurance	Empathy
Binary relevance	NB	Accuracy	0.675	0.690	0.636	0.643	0.782
		Recall	0.271	0.998	0.390	0.797	1.000
		Precision	0.765	0.689	0.665	0.603	0.782
		F1-score	0.399	0.815	0.485	0.681	0.878
	SVM	Accuracy	0.716	0.736	0.640	0.730	0.786
		Recall	0.511	0.885	0.514	0.730	0.951
		Precision	0.692	0.765	0.619	0.719	0.809
		F1-score	0.587	0.820	0.558	0.721	0.874
	LR	Accuracy	0.680	0.715	0.657	0.733	0.792
		Recall	0.369	0.970	0.464	0.764	0.999
		Precision	0.678	0.716	0.675	0.711	0.791
		F1-score	0.474	0.823	0.546	0.732	0.883
Label powerset	NB	Accuracy	0.661	0.692	0.554	0.566	0.782
		Recall	0.497	0.998	0.876	0.941	0.999
		Precision	0.612	0.690	0.506	0.529	0.783
		F1-score	0.531	0.816	0.633	0.675	0.878
	SVM	Accuracy	0.666	0.685	0.610	0.636	0.787
		Recall	0.471	0.884	0.688	0.816	0.948
		Precision	0.618	0.720	0.553	0.590	0.812
		F1-score	0.527	0.793	0.610	0.682	0.874
	LR	Accuracy	0.642	0.702	0.614	0.612	0.802
		Recall	0.429	0.941	0.738	0.825	0.980
		Precision	0.576	0.714	0.555	0.567	0.808
		F1-score	0.487	0.812	0.629	0.670	0.886
Classifier chain	NB	Accuracy	0.675	0.690	0.635	0.652	0.782
		Recall	0.271	0.997	0.371	0.786	1.000
		Precision	0.765	0.689	0.675	0.619	0.782
		F1-score	0.399	0.814	0.473	0.684	0.878
	SVM	Accuracy	0.716	0.731	0.651	0.737	0.799
		Recall	0.511	0.873	0.538	0.730	0.938
		Precision	0.692	0.766	0.630	0.727	0.829
		F1-score	0.587	0.816	0.577	0.726	0.879
	LR	Accuracy	0.680	0.716	0.644	0.716	0.794
		Recall	0.369	0.961	0.546	0.706	0.977
		Precision	0.678	0.719	0.617	0.713	0.803
		F1-score	0.474	0.822	0.576	0.704	0.881
RakEL	NB	Accuracy	0.639	0.692	0.628	0.648	0.782
		Recall	0.173	0.995	0.506	0.714	1.000
		Precision	0.689	0.691	0.651	0.630	0.782
		F1-score	0.274	0.815	0.521	0.657	0.878
	SVM	Accuracy	0.717	0.707	0.630	0.688	0.785
		Recall	0.494	0.900	0.522	0.719	0.952
		Precision	0.708	0.733	0.598	0.666	0.807
		F1-score	0.580	0.808	0.555	0.688	0.874
	LR	Accuracy	0.675	0.718	0.650	0.693	0.799
		Recall	0.396	0.931	0.521	0.721	0.983
		Precision	0.654	0.732	0.641	0.679	0.804
		F1-score	0.491	0.819	0.563	0.693	0.884
MLkNN	N/A	Accuracy	0.648	0.688	0.629	0.641	0.761
	N/A	Recall	0.493	0.829	0.530	0.683	0.936
	N/A	Precision	0.565	0.745	0.600	0.616	0.795
	N/A	F1-score	0.526	0.783	0.554	0.645	0.859
BRkNN	N/A	Accuracy	0.640	0.690	0.641	0.631	0.750
	N/A	Recall	0.292	0.860	0.376	0.529	0.878
	N/A	Precision	0.614	0.734	0.689	0.645	0.817
	N/A	F1-score	0.388	0.790	0.479	0.580	0.844

**Table 3:** Performance metrics of MLSA with 5-fold cross validation.

Model	Accuracy	Recall	Precision	F1-Score
NB	0.7810	0.9988	0.7769	0.8740
SVM	0.8743	0.9363	0.9028	0.9189
LR	0.8429	0.9917	0.8334	0.9057

**Table 4:** Performance metrics of MLSA with hold out method.

Model		Accuracy	Recall	Precision	F1-Score
NB	Negative	81%	19%	100%	33%
	Positive		100%	80%	89%
SVM	Negative	90%	73%	82%	77%
	Positive		95%	92%	93%
LR	Negative	87%	49%	92%	64%
	Positive		99%	86%	92%

## Data Availability

The Facebook data presented in this study are available on request from the corresponding author. The data are not publicly available due to privacy and confidentiality. However, restrictions apply to the availability of hospital data. The data is available from the authors with permission of Ministry of Health.

## Appendix A

**Table A1:** SERVQUAL Guideline.

Domain	Description	Facebook Reviews Example
Tangible	General: The appearance of employees, equipment, and physical facilities of the hospital.	“Cleanliness of the Hospital is good”
	Specific: The hospitals have up to date equipment. The physical facilities are visually new or outdated. The staffs are well dressed, appear neat and good looking. The appearance of the physical facilities of the hospital are well maintained with the type of services provided.	“Car parking is difficult and limited”
		“Satisfied with the facilities. Large room, feels like a hotel.”
		“The hospital is well maintained, and their food is delicious.”
Reliability	General: Accurate, dependable, and consistent performance of the service.	“My appointment scheduled at 9 am but then it was postponed to 12.00 pm. Unbelievable.”
	Specific: When the hospital promised to do something by a certain time, it does so. Hospital service is efficient and dependable. The hospital provides services at the time as promise to do so. The hospital keeps the records accurately or at online.	“System needs to be improved especially discharge process. It took hours to settle it.”
		“Efficient and top-quality hospital services”
		“Staff mistakenly collected medical record of other patient with similar name of mine”
Responsiveness	General: Willingness to provide prompt service to the patients.	“My specialist took his time to explain me about my disease and how he will treat it”
	Specific: The hospital let patients know exactly when the services will be performed. The staffs give prompt services to patients upon request. The staffs are always willing to help their patients. The staffs give medical attention promptly.	“They answered all my questions during the admission.”
		“Arrived at emergency department due to road traffic accident and the medical team immediately respond to it.”
		“I don’t feel any pain throughout the minor surgery on my arm, and it was done in a flash”
Assurance	General: the staff knowledge and courtesy, ability to inspire trust, confidence, and security. Also reflects on confidentiality and privacy of patients.	“The surgery was successful. Mr A is a competent and trusted surgeon.”
	Specific: The staffs are trustworthy. Patients feel safe in their transactions with the hospitals. The staffs are polite, friendly. The staffs have adequate support from the hospitals to do their jobs well.	“I feel comfortable and safe in this hospital. Just like at home”
		“The staff at the front desk was rude.”
		“The doctors and staff nurses in this hospital are skillful and well-trained”
Empathy	General: Providing convenient services and giving attention or patience of the staffs to the patients’ needs.	“Nurses are very helpful.”
	Specific: The staffs give patient personal attention and helpful. The staffs are knowledgeable to understand patient’s specific needs. The hospital has patient best interests at heart. The hospital has operating hours convenient to all the patients. Cost of treatment is affordable for patients	“A staff came and offered to help my father climb stairs without we ask him. We appreciated his kindness.”
		“They are very concerned about patient’s condition and served it with their heart”
		“The price is affordable compared to private hospital.”

**Table A2:** Sentiment Analysis Guideline.

Category	Description	Facebook Reviews Example
Positive	Expression of liking, approval, gratefulness	“I like this hospital. Doctors and nurses are pleasant and helpful.”
	(Like, love, support, thankful etc.)	“Thank you for your service, Doctor and nurses.”
	Positive qualities of hospital services and facilities	“The wait time was brief. The pharmacy counter did an excellent job.”
	(Clean room, efficient, fast appointment, affordable etc.)	“The room is neat and tidy, and the food is delicious. I really like it.”
	Positive qualities of staff	“Staff are polite and kind.”
	(Polite, friendly, helpful, responsive etc.)	“Dr. B took her time explaining my health condition until I understood it. It was greatly appreciated.”
	Encourage or recommend others to use	“I recommend having your baby delivered at this hospital.”
		“I like their antenatal counselling and will recommend it to other couples. It is extremely beneficial to us.”
	Positive/desirable effects of service	“I’d like to thank Mr A for performing bowel surgery on my father. He is now doing well.”
	(Successful treatment/procedures, good health outcome etc.)	“I found the physiotherapy session to be beneficial. I’m able to walk with less pain now.”
Negative	Expression of disliking or disapproval	“I hate the security guard.” He was impolite to me!”
	(Do not like, hate etc.)	“I’m not a fan of the food service here. The food has no taste.”
	Negative characteristic of hospital services or facilities (Poor maintenance, slow service, expensive, long waiting time etc.)	“The discharge procedure was extremely slow.”
		“There are a limited number of parking spaces available, and getting one is difficult.”
		“We waited for 5 h at the out-patient clinic before seeing the doctor. This is intolerable.”
	Negative qualities of staff	“Staff nurses were rude and stubborn. I requested assistance but received no response.”
	(Rude, not-friendly, not-helpful, slow responsive, incompetency etc.)	“The doctor criticised us for arriving at the emergency department at 3 a.m. for treatment. We were annoyed by his attitude.”
	Negative/undesirable effects	“My father fell in the toilet and was left alone for a few minutes. The hospital director must explain the incident to our family.”
	(Surgical or procedural complications, medicolegal, poor health outcome etc.)	“After being admitted to this hospital two days ago, my husband’s condition has deteriorated. No one, however, can explain the situation to us.”
Neutral	Review that reports factual	“Serdang Hospital is one of the Klang Valley’s cardiac centres.”
	information/no opinion.	“A Muslim-friendly hospital”
	Review as questions	“Do you have any spine surgeon in your hospital?”
		“How to get an appointment with your ear. Nose and throat (ENT) clinic?”
	Too ambiguous/unclear/Greetings only	“Good morning.”
		“No comment.”
		“Let’s wait and see first”
